# A systematic review of scope and quality of health economic evaluations conducted in Ethiopia

**DOI:** 10.1093/heapol/czac005

**Published:** 2022-02-21

**Authors:** Daniel Erku, Amanual G Mersha, Eskinder Eshetu Ali, Gebremedhin B Gebretekle, Befikadu L Wubishet, Gizat Molla Kassie, Anwar Mulugeta, Alemayehu B Mekonnen, Tesfahun C Eshetie, Paul Scuffham

**Affiliations:** Centre for Applied Health Economics, Griffith University, Nathan, QLD, Australia; Menzies Health Institute Queensland, Griffith University, Gold Coast, QLD, Australia; Addis Consortium for Health Economics and Outcomes Research (AnCHOR); School of Medicine and Public Health, The University of Newcastle, Newcastle, Australia; Department of Pharmaceutics and Social Pharmacy, School of Pharmacy, College of Health Sciences, Addis Ababa University 251, Yared Street, Tikur Anbessa Specialized Hospital, Addis Ababa, Ethiopia; Institute of Health Policy, Management, and Evaluation, University of Toronto, 155 College Street, Toronto, Ontario M5T 3M6, Canada; Toronto Health Economics and Technology Assessment (THETA) Collaborative, University Health Network, 200 Elizabeth Street, Toronto, ON M5G 2C4, Canada; Health Services Research Centre Faculty of Medicine, The University of Queensland, 288 Herston Road, Herston, 4006, Brisbane, Australia; Quality Use of Medicines and Pharmacy Research Centre, University of South Australia: Clinical & Health Sciences, Adelaide, SA 5000, Australia; Australian Centre for Precision Health, Unit of Clinical and Health Sciences, University of South Australia, Adelaide, SA 5000, Australia; Department of Pharmacology and Clinical Pharmacy, College of Health Sciences, Addis Ababa University, Addis Ababa 1000, Ethiopia; South Australian Health and Medical Research Institute, Adelaide, SA 5000, Australia; Centre for Quality and Patient Safety Research, School of Nursing and Midwifery, Institute for Health Transformation, Deakin University, 221 Burwood Highway, Burwood, VIC 3125, Australia; Plein Center for Geriatric Pharmacy Research, Education and Outreach, School of Pharmacy, University of Washington, 1959 NE Pacific Street, Seattle, WA 98195-7630, USA; Centre for Applied Health Economics, Griffith University, Nathan, QLD, Australia; Menzies Health Institute Queensland, Griffith University, Gold Coast, QLD, Australia

**Keywords:** Ethiopia, economic evaluation, cost-effectiveness

## Abstract

There has been an increased interest in health technology assessment and economic evaluations for health policy in Ethiopia over the last few years. In this systematic review, we examined the scope and quality of healthcare economic evaluation studies in Ethiopia. We searched seven electronic databases (PubMed/MEDLINE, EMBASE, PsycINFO, CINHAL, Econlit, York CRD databases and CEA Tufts) from inception to May 2021 to identify published full health economic evaluations of a health-related intervention or programme in Ethiopia. This was supplemented with forward and backward citation searches of included articles, manual search of key government websites, the Disease Control Priorities-Ethiopia project and WHO-CHOICE programme. The quality of reporting of economic evaluations was assessed using the Consolidated Health Economic Evaluation Reporting Standards (CHEERS) checklist. The extracted data were grouped into subcategories based on the subject of the economic evaluation, organized into tables and reported narratively. This review identified 34 full economic evaluations conducted between 2009 and 2021. Around 14 (41%) of studies focussed on health service delivery, 8 (24%) on pharmaceuticals, vaccines and devices, and 4 (12%) on public-health programmes. The interventions were mostly preventive in nature and focussed on communicable diseases (*n* = 19; 56%) and maternal and child health (*n* = 6; 18%). Cost-effectiveness ratios varied widely from cost-saving to more than US $37 313 per life saved depending on the setting, perspectives, types of interventions and disease conditions. While the overall quality of included studies was judged as moderate (meeting 69% of CHEERS checklist), only four out of 27 cost-effectiveness studies characterized heterogeneity. There is a need for building local technical capacity to enhance the design, conduct and reporting of health economic evaluations in Ethiopia.

Key messages

- There has been an increased interest in the use of health economic evaluations in health policymaking in Ethiopia over the last few years.
- This paper comprehensively examined the methodological rigour and reporting of published health economic evaluations in Ethiopia.
- The number and scope of health economics evaluations conducted in Ethiopia are severely limited, with only a handful of articles conducted on non-communicable diseases.
- There is a need to boost funding and build local technical capacity for conducting high-quality and policy-relevant health economic evaluations.

## Background

Universal health coverage (UHC) is realized when all people have access to quality essential healthcare services when and where they need them, without financial hardship [World Health Organization (WHO), 2020]. Many countries across the globe have reaffirmed their commitments to achieve UHC by 2030 (WHO, 2020). However, meeting this target is evidently difficult for many low- and middle-income countries (LMICs) where out-of-pocket (OOP) health expenditure is still the major source of financing health services. In this regard, Ethiopia is not an exception in that OOP puts a significant hurdle on accessibility of health services to the poor (Kiros *et al.*, 2020). To overcome this, the Ethiopian government recently introduced equity-driven health insurance schemes that gives due consideration to the economically disadvantaged segments of the society including the agricultural and informal sectors (Mebratie *et al.*, 2015).

With the increasing insurance coverage comes the need for a systematic approach of selecting the most cost-effective healthcare technologies and interventions for healthcare providers and governments to invest in and/or reimburse. This requires the application of explicit sets of criteria—often in the form of health technology assessment (HTA)—to systematically synthesize evidence on clinical and cost-effectiveness as well as budget impact of health technologies (O’Rourke *et al.*, 2020). An integral component of HTA is health economic evaluation which involves generating and integrating evidence on clinical, economic and patient-reported outcomes to determine whether patients are likely to benefit from health technologies at acceptable cost to the healthcare system (Drummond *et al.*, 2015).

Despite the relevance and established role of HTA in healthcare decision-making in developed countries, it has not been effectively implemented in many LMICs (Zegeye *et al.*, 2018). The main reasons for this are lack of local data, limited technical expertise, reliance on traditional decision-making and lack of formal HTA institutions (Babigumira *et al.*, 2016; Zegeye *et al.*, 2018). Nonetheless, there has been an increased interest in the use of health economic evaluations in health policymaking in Ethiopia over the last few years (Zegeye *et al.*, 2018). The government’s most notable initiative is the recent establishment of a Health Economics and Financing Analysis unit within the Ministry of Health with the aim of conducting policy-relevant HTA on programmes, procedures and treatments (Zegeye *et al.*, 2018). The increased interest in HTA is also reflected in the recent increase in the number of publications related to health economic evaluations in Ethiopia (Zegeye *et al.*, 2018). However, little is known about the scope, quantity and quality (i.e. methodological rigour and reporting) of these economic evaluations. In this systematic review, we examined the scope and quality of health economic evaluation studies in Ethiopia. Specifically, we examined the number, quality, methodology and focus of published healthcare economic evaluation studies and explored the range of willingness to pay thresholds used to determine cost-effectiveness of interventions.

## Methods

The review was conducted following the International Society for Pharmacoeconomics and Outcomes Research (ISPOR) Criteria for Cost-Effectiveness Review Outcomes Checklist (Mandrik *et al.*, 2021) and the Preferred Reporting Items for Systematic Reviews and Meta-Analyses (PRISMA) guideline (Moher *et al.*, 2009). We followed van Mastrigt’s five-step approach for conducting systematic reviews of economic evidence (van Mastrigt *et al.*, 2016), and the review protocol was registered on PROSPERO.

### Search strategy

We searched seven electronic databases [PubMed/MEDLINE, EMBASE, PsycINFO, CINHAL, Econlit, York CRD databases (Database of Abstracts of Reviews of Effects and the NHS Economic Evaluation Database) and CEA Tufts] for published health economic evaluations of a health-related intervention or programme in Ethiopia. We considered all articles published from inception of each database to May 2021 to search, without any restriction on year of publication. We complemented the database searches with forward and backward reference searching. In addition, we searched grey literature including (1) manual search of key government websites, (2) the Disease Control Priorities-Ethiopia (DCP-E) project (DCP-3, 2020) and (3) WHO-CHOICE (CHOosing Interventions that are Cost-Effective) programme World Health Organisaion (WHO), Cost Effeciveness and Strategic Planning (WHO-CHOICE).

The keywords used in the optimized search strategy were built on two key concepts of the subject as (1) health economic evaluations (‘Health Economics’ OR ‘Economics, Hospital’ OR ‘Economics, Medical’ OR ‘Economics, Nursing’ OR ‘Economics, Pharmaceutical’ OR ‘Economics’ OR ‘economic evaluation’ OR ‘economic model’ OR ‘costs and cost analysis’ OR ‘Cost-Benefit Analyses’ OR ‘Cost Benefit Analysis’ OR ‘Cost utility’ OR ‘Cost effectiveness’ OR ‘Cost-effectiveness’ OR ‘Cost-benefit’, ‘pharmacoeconomics’ OR ‘health technology assessment’) and (2) Ethiopia (‘Ethiopia’ OR ‘Ethiopian’ OR ‘Ethiop*’) and tailored to each database. We combined subject headings and free text terms with various Boolean operators and truncations tailored to each database.

### Eligibility screening

**Figure 1.**
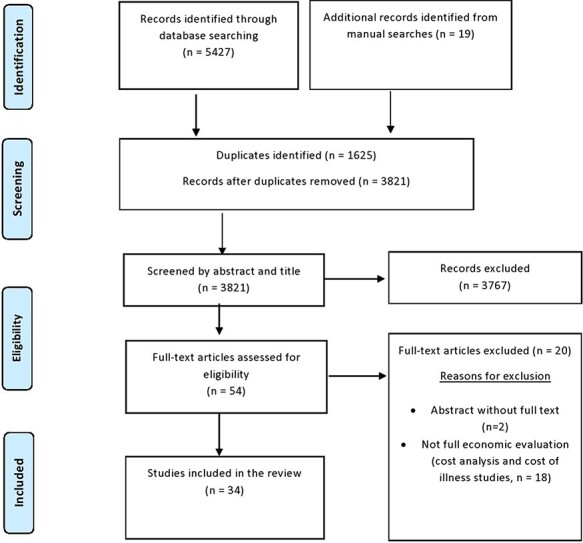
PRISMA flow diagram

We included full health economic evaluations (comparing costs and outcomes of two or more healthcare interventions) and cost–outcome descriptions of health technologies conducted in an Ethiopian setting. Studies were considered full health economic evaluations if they compared costs and consequences of two or more healthcare interventions alternatives, including cost–consequences analysis, cost-minimization analysis, cost-effectiveness analysis, cost–utility analysis and cost–benefit analysis. Health technology is defined by WHO as ‘the application of organized knowledge and skills in the form of medicines, medical devices, vaccines, procedures and systems developed to solve a health problem and improve quality of life (World Health Organisation (WHO))’. Except for articles that described costs and outcomes of a single service or programme (i.e. cost–outcome descriptions), all other types of partial health economic evaluations (i.e. cost description, outcome description, cost analysis and cost of illness studies) were excluded. We also excluded methodology papers, literature reviews and conference or dissertation abstracts without the full text available for retrieval. Prior to excluding conference abstracts, dissertation abstracts and other relevant articles without full text, a repeated email contact was made with authors requesting for full text. Two authors independently screened titles, abstracts and full texts of articles in COVIDENCE based on the eligibility criteria. Any differences between the two reviewers were resolved by consensus, and if necessary, with the help of a third reviewer. The detailed search strategy and eligibility screening are summarized in Figure 1.

### Reporting quality of studies

We employed the Consolidated Health Economic Evaluation Reporting Standards (CHEERS) checklist (Husereau *et al.*, 2013) to assess reporting quality of included studies. CHEERS tool was employed for this review to help interpret the findings; i.e. to ascertain the quality of evidence from the included articles, but studies were not excluded based on quality. The CHEERS checklist was developed by ISPOR Task Force with the aim of providing a guidance for researchers, editors and peer reviewers regarding optimal reporting of health economic evaluations. The checklist consists of 24 items subdivided into six main categories: (1) title and abstract, (2) introduction, (3) methods, (4) results, (5) discussion and (6) ‘other’. Studies were scored independently by two of the authors, in consultation with a third reviewer (AM) as having met the criteria in full (designated as ‘Yes’ and given a score of 1), do not fulfil (designated as ‘No’ and given a score of 0) or not applicable (‘NA’).

### Data extraction and synthesis

Two of the authors independently extracted data from a random sample of articles (20%) using a published data extraction form, after tailoring to our review objective and the study designs of included articles (Wijnen *et al.*, 2016). Once agreement was reached between the two reviewers on the content and method of data extraction (via pilot testing with three randomly selected studies), DE undertook data extraction from the remaining studies and AGM independently cross-checked the data against the original studies. The data extraction form included two main sections: (1) study characteristics (e.g. publication details, country, study design, sample size, intervention/comparator, study perspective, analytical approach, etc.) and (2) study design and main outcomes [resource use, costs, effects, measurement, valuation methods if applicable, total and incremental quality adjusted life years (QALYs), incremental cost-effectiveness ratios (ICER) and author’s conclusions]. For the sake of clarity and improving comparability among studies, we classified health technologies into ‘pharmaceuticals, vaccines and devices’, ‘health service delivery including procedures’, ‘public health, health promotion or prevention programs’ or ‘multiple’ (a combination of the above). Data were also extracted on key model parameters reported in the sensitivity analyses (if any), along with their impact on the overall ICER estimate. The extracted data were grouped into subcategories based on the subject of the economic evaluation, organized into tables and reported narratively.

## Results

### Characteristics of included studies

**Figure 2.**
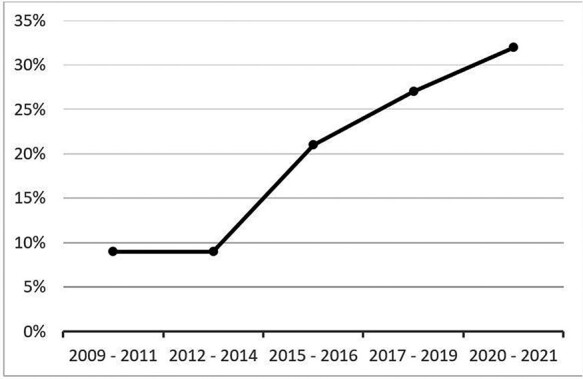
Number of publications according to year, *N* = 34

After title, abstract and full-text screening, a total of 34 full economic evaluations were included in this systematic review (27 cost-effectiveness analysis (Pecenka *et al.*, 2015; Bikilla *et al.*, 2009; Datiko and Lindtjørn 2010; Lemma *et al.*, 2011; Curry *et al.*, 2013; Johns *et al.*, 2014; McPake *et al.*, 2015; Strand *et al.*, 2016; Tolla *et al.*, 2016; Accorsi *et al.*, 2017; Kolesar *et al.*, 2017; Mathewos *et al.*, 2017; Adelman *et al.*, 2017; Hailu *et al.*, 2018; Hounsome *et al.*, 2019; Kebede *et al.*, 2019; Memirie *et al.*, 2019; Yigezu *et al.*, 2020; Carvalho *et al.*, 2020; Cha *et al.*, 2020; Devine *et al.*, 2020; Madan *et al.*, 2020; Alemayehu *et al.*, 2020; Assebe *et al.*, 2021; Belay *et al.*, 2021; Crocker *et al.*, 2021; Eregata *et al.*, 2021; Olsen *et al.*, 2021) and 7 extended cost-effectiveness analysis (Verguet *et al.*, 2013; Driessen *et al.*, 2015; Johansson *et al.*, 2015; 2015; Shrime *et al.*, 2016; Johansson *et al.*, 2017; Assebe *et al.*, 2020) (Figure 1). There has been a significant increase in the number of published full economic evaluations in Ethiopia in the past five years (only six publications between 2009 and 2014 compared with 20 publications between 2017 and 2021) (Figure 2). Half of the studies were funded either by multiple international donors (*n* = 9; 26%) or United Nations/bilateral aid agencies (*n* = 8; 24%) whereas the remaining were funded by research institutions or universities (*n* = 7; 21%) and the government (*n* = 4; 12%) (Table 1). Nearly all of the studies (*n* = 32; 96%) were conducted in collaboration with researchers outside Ethiopia, with at least one co-author affiliated with a foreign institution. Majority of the included studies were identified as cost-effectiveness analysis and involved several disease conditions (Table 1). The interventions were mostly preventive in nature and focussed on communicable diseases (56%) or maternal and child health (18%), with only one study conducted on cardiovascular disease (Tolla *et al.*, 2016) and two on mental health (Strand *et al.*, 2016; Johansson *et al.*, 2017). Around 35% of studies focussed on health service delivery, 29% on procedure, medications and devices, and 12% on public-health programmes.

**Table 1.** Description of included studies, *N* = 34

Category	Description	*N* (%)
Type of health technology	Health service delivery	14 (41)
	Pharmaceuticals, vaccines and devices	8 (24)
	Public health, health promotion or prevention programmes	4 (12)
	Multiple (two or more of the above)	8 (24)
Medical condition	Tuberculosis and/or HIV/AIDS	9 (26)
	Maternal and child health	6 (18)
	Malaria	4 (12)
	Podoconiosis	1 (3)
	Measles	1 (3)
	Pneumonia	2 (6)
	Diarrhoea	1 (3)
	Cardiovascular disease	1 (3)
	Mental health	2 (6)
	Multiple	7 (21)
Study design	Trial based	4 (12)
	Modelling—Markov model	5 (15)
	Modelling—decision analytic model	6 (18)
	Modelling—WHO-CHOICE tools	8 (12)
	Mixed (Trial based plus modelling)	2 (6)
	Extended cost-effectiveness analysis	7 (21)
	Cost–outcome description	1 (3)
Study perspective	Provider/payer	19 (56)
	Societal	6 (18)
	Patient	1 (3)
	Not clear	7 (21)
Time horizon	Less than a year	1 (3)
	1–10 years	15 (44)
	>10 years but not lifetime	3 (9)
	Lifetime	4 (12)
	Not mentioned (not clear)	11 (32)
Discount rate used	3%	24 (71)
	>3%	2 (6)
	Not applied	1 (3)
	Not reported (not clear)	7 (20)
Outcome measure^a^	Disability adjusted life years (DALYs)	10 (29)
	Health life year gained	8 (24)
	Clinical and/or patient-reported end points	3 (9)
	Deaths averted	8 (24)
	Household OOP expenditures averted and expected financial risk protection	7 (21)
	QALYs	1 (3)
	Not mentioned (not clear)	4 (12)
Sources of funding	Government	4 (12)
	Non-governmental organizations	8 (24)
	Research institution (university)	7 (21)
	Multiple	9 (26)
	Consultancy	1 (3)
	Not funded	1 (3)
	Not mentioned	3 (9)

a More than one outcome measure may be reported.

### Assessment of the reporting quality of studies

The assessment of the reporting quality of each study using the CHEERS checklist is summarized in Supplementary Figure S1. Overall, the reporting quality of the included studies varied from 40% to 84% (median 69%) on the CHEERS criteria. The most consistently reported elements were title, abstract, background and objectives, target population, setting, comparators, measurement of effectiveness, choice of health outcomes, analytic methods, sources of funding, conflicts of interest and study limitations, with 95% or more of studies reporting these elements. While all model-based studies explicitly stated the modelling approach, none of them gave reasons for the specific type of model employed. The item that least complied with the CHEERS was on characterizing heterogeneity, compliant only in 4 out of 27 cost-effectiveness articles (excluding extended cost-effectiveness analysis articles).

About two-third of the studies explicitly reported the perspective of economic evaluation, the most common being healthcare provider (56%) followed by societal perspective (18%). All studies included ‘standard of care (SoC)’ or the routine programme or care as a comparator. The time horizons for the evaluations ranged from three months (Accorsi *et al.*, 2017) to lifetime (Hailu *et al.*, 2018; Yigezu *et al.*, 2020; Carvalho *et al.*, 2020; Olsen *et al.*, 2021). The discount rates used were reported in 26 (74%) of the studies, out of which 24 (92%) used a 3% discount rate. For the remaining studies, discounting was either not reported (*n* = 7) or not applicable as the main analysis considered a time horizon of less than 12 months (*n* = 1).

### Reporting of costs and health outcomes

All except two studies (Curry *et al.*, 2013; Eregata *et al.*, 2021) described the approach used to estimate unit costs and cost calculations. Several sources were used to derive data regarding costing of resource use including from literature reviews (e.g. previous economic evaluations and resource utilization study) (*n* = 26; 74%) and/or expert opinion (*n* = 9; 26%). While the types of costs included depended on the study setting and study perspectives, drug costs, direct medical costs (e.g. laboratory tests) and health system or hospital-related costs (e.g. homecare workers and general practitioners) were the key inputs for the cost analysis in majority of the studies. Among studies that reported the source of resource utilization data (*n* = 32), 14 (44%) of them incorporated data from a study conducted in Ethiopia whereas the remaining studies utilized data from developed countries.

All studies clearly described the choice of outcomes. Outcome measures for consequences or benefits were DALYs (*n* = 10; 29%), life years gained (*n* = 8; 24%) and deaths averted (*n* = 8; 24%). Only one study used QALYs as the summary health outcome measure (Belay *et al.*, 2021). Economic modelling was used by 19 studies to generate costs and benefits (five Markov, six decision analytic tree and WHO-CHOICE tools). Four studies conducted their evaluation alongside observational trials and two studies used routinely collected patient data. Input parameters for model-based studies were retrieved from several sources. The Global Burden of Disease and Demographic Health Survey were the most common source of epidemiological data (*n* = 26; 76%). Most of the effectiveness data were estimated from randomized controlled trials (RCTs) (28%) and observational data (20%) or were estimated based on a literature review.

### Cost-effectiveness outcomes according to disease conditions

More than half (*n* = 15, 56%) of the 27 cost-effectiveness studies reported ICERs as the final economic evaluation outcome. All except six studies (Datiko and Lindtjørn 2010; Lemma *et al.*, 2011; Johns *et al.*, 2014; Hounsome *et al.*, 2019; Cha *et al.*, 2020; Crocker *et al.*, 2021) clearly stated the willingness to pay (WTP) threshold used, of which 78% referred to the WHO-CHOICE framework to determine cost-effectiveness. Cost-effectiveness ratios varied widely from cost-saving (Hounsome *et al.*, 2019) to more than US $37 313 per life saved (Curry *et al.*, 2013) depending on the setting, perspectives, types of interventions and disease conditions. Details of cost-effectiveness outcomes according to disease conditions are summarized in Supplementary Tables S2 and S3. Majority of the studies (*n* = 31; 91%) conducted sensitivity analysis. Around half of the studies (*n* = 15; 54%) reported performing probabilistic sensitivity analysis, while three studies conducted bootstrapping. Parameters with the greatest influence on model outcomes were uncertainties in epidemiological data, intervention costs and other costs such as costs of hospitalizations.

## Discussion

The use of a systematic approach (such as via HTA) to allocate scarce healthcare resources in Ethiopia and other LMICs has become important than ever before with the current global economic crisis caused by the COVID-19 pandemic, and the substantial reduction in external funding. Our review indicates that health economic research in Ethiopia is at an early stage of development, with only 34 full health economic evaluations identified, but have increased from 3 in 2013/14 to 11 in 2020/21. Previous systematic reviews reported a similar or slightly higher number of economic evaluations conducted in other sub-Saharan Africa countries (Panzer *et al.*, 2020) [e.g. 45 in South Africa (Gavaza *et al.*, 2012), 44 in Nigeria (Gavaza *et al.*, 2010)]. This can partially be explained by the lack of technical capacity to undertake and/or interpret economic evaluations and lack of adequate technical knowledge on health economics and HTA across the health sector (Zegeye *et al.*, 2017).

Our findings revealed a severe lack of local research funding for conducting health economic evaluations, with only 12% of the studies funded by the government. More than half of studies were funded by NGOs or foreign institutions and focussed mainly on communicable disease and/or maternal and child health. There were only a few (*n* = 3) of health economic evaluations in non-communicable diseases. While infectious diseases and maternal and child health are still the major causes of mortality and morbidity, Ethiopia is also experiencing a substantial rise in the prevalence of major non-communicable diseases such as cardiovascular disease, cancer, diabetes mellitus and chronic obstructive pulmonary disease (Shiferaw *et al.*, 2018; Girum *et al.*, 2020). Thus, the economic evaluations published so far may not reflect Ethiopia’s current disease burden. Thus, there is a need for more funding, both from the government and external funding agencies, to generate reliable, policy-relevant economic evidence in major chronic diseases.

The majority of the studies evaluated interventions related to health service delivery or public health, health promotion or prevention programmes. There are several challenges when conducting cost-effectiveness of public health interventions including long-term impact of interventions, identifying costs and consequences which are often complex and intersectoral in nature, incorporating equity considerations, and difficulty of undertaking RCTs for comparing relevant alternatives. When conducting cost-effectiveness of public health interventions, the comparators are interventions routinely delivered in the community, which are often one i.e. regarded as ‘best practice’ (Kelly, 2010). However, studies that report ‘best practice’ as a comparator may have different definitions and/or understandings of what ‘best practice’ constitutes. Thus, such factors need to be considered by decision makers, along with the relevant costs and benefits, when deciding which interventions to fund.

All of the studies included in this review are either cost-effectiveness analysis (CEA) or extended cost-effectiveness analysis (ECEA) in design. A similar pattern has also been reported in other LMICs (Teerawattananon *et al.*, 2007; Tran *et al.*, 2014; Prinja *et al.*, 2015). ECEA is an extension of CEA and is contextualized to specific health system setting with a major emphasis to equity, distributional health consequence and financial risk protection. Developed for DCP3 programme, ECEA has been instrumental in directly informing several health policies in many LMICs. Ethiopia has also greatly benefited from the DCP3 programme and ECEA method when revising the country’s Essential Health Service Package in 2019 (Eregata *et al.*, 2020). The method is relatively new and as such the qualities of these studies (both content and reporting wise) were not examined given the lack of standardized tool for ECEA and the difficulty of applying existing tools (such as CHEERS).

It is also worth mentioning that only one cost–utility study was identified in our review (Belay *et al.*, 2021). The methodological complexity of computing QALY and lack of local utility weights for common quality of life instruments may partially explain the dearth in cost–utility analyses. A group of researchers recently developed a value set for the European Quality of Life Five Dimension, Five Level (EQ-5D-5L) using an Ethiopian general population sample (Welie *et al.*, 2020). Being the first EQ-5D-5L valuation study in Africa, it is expected that the availability of this value set will facilitate cost–utility analysis and inform policy decision-making in Ethiopia.

Among the studies that clearly stated the WTP threshold was used, 78% of studies referred to the WHO-CHOICE framework to determine cost-effectiveness. WHO’s CHOICE recommends project the WTP as 1–3 times the country’s annual gross domestic product (GDP) per capita per DALYs averted. Several concerns have been raised regarding the usefulness of this approach in decision-making (Marseille *et al.*, 2014). Specifically, this approach lacks a firm theoretical justification and assumptions and obscures fundamental comparisons from the equation. Knowing a particular health technology costs less than three times the country’s annual GDP per capita per DALYs averted is not enough as its relative value should also be compared against (1) other essential and feasible health interventions and (2) the country’s health budget (an intervention i.e. ‘cost-effective’ still may not represent the best use of a country’s health budget). This coupled with the easy attainability of this threshold by too many interventions without a critical appraisal of affordability (and trade-offs) makes the threshold not fit for purpose. For economic evaluation to contribute to healthcare decision-making, all estimates and assumptions (i.e. costs and outcomes) should be contextualized with the local context (budget, disease burden, etc.). As such, there is a need for a WTP threshold i.e. reflective of the local contexts rather than depending on a potentially misleading, one-size-fits-all threshold.

In 2015, the Federal Ministry of Health of Ethiopia established the Health Economics and Financing Analysis (HEFA) unit to provide high-level advice on HTA for the top ministerial management office (Zegeye *et al.*, 2018). The establishment of HEFA enhanced the technical capacity and increased awareness among policymakers. This is clearly reflected in the government’s use of health economic evidence as one of the essential criteria (within the broader Multi Criteria Decision Analysis—MCDA approach) when revising its Essential Health Service Package in 2019 (Eregata *et al.*, 2020). The use of MCDA in decision-making and consideration of evidence from economic evaluations is an indication of the growing interest in and use of evidence to inform health policies. However, the lack of contextualized cost-effectiveness analyses means that such decision-making processes depend mainly on evidence synthesis, supplemented with limited local evidence. Future economic evaluations and fiscal space analyses should use, where possible, country-specific data costs, epidemiology, demography, baseline coverage or effects. This will not only produce actionable and readily interpretable evidence to policymakers, but also reduce reliance on economic evaluations conducted in a different health system from which transferability of cost-effectiveness ratios is more challenging. The recent expansion in graduate-level health economics education in Ethiopia, if matched with necessary research funding, is expected to create the critical mass of researchers and enhance the number of high-quality research output necessary for the efficient allocation of resources in the country’s health sector.

Findings from assessment of reporting quality of studies revealed some areas of improvement for conduct and reporting of cost-effectiveness studies. These include adequately describing target population and ‘usual care’ comparators (including description of what this entails), identifying, measuring and valuing all relevant costs, adequately describing analytical methods, and accounting for uncertainty and characterizing heterogeneity. Strengthening the quality of health economic studies through measures such as training and capacity building will increase the quality, credibility and uptake of such evidence.

### Strength and limitations

This review is the first attempt in Ethiopia to map the scope and quality of healthcare economic evaluations conducted in Ethiopia. While we employed a robust search strategy and accepted methods to retrieve and present the data, we may have missed some health economic evaluations, particularly those that are conducted as part of a HTA report as these are often not indexed in electronic databases. The inherent subjectivity of assessing the reporting quality of included studies (Watts and Li 2019) is another key limitation of this review although we have used a second reviewer and a consensus process to reduce the subjectivity in scoring CHEERS checklists. Nonetheless, this review highlighted the major gaps in the literature, summarized the qualities of published health economic studies and pinpointed areas in which more evidence is needed.

## Conclusions

Our review indicated that full health economic evaluations are sparce in Ethiopia, particularly in non-communicable disease areas, and many of the identified studies were not of high quality. The scope, content or quality of currently available evidence base is inadequate to reliably inform sound policymaking. There is a need to boost funding and build local technical capacity for conducting high-quality HTA and health economic evaluations that will help inform priority-setting activities including regulatory, coverage/formulary and reimbursement policy decisions.

## Supplementary Material

czac005_Supp

## Data Availability

The data underlying this article are available in the article and in its online supplementary material.
