# Supplementary material for: A systematic review of scope and quality of health economic evaluations conducted in Ethiopia
Source: Health Policy Plan. 2022 Feb 21;37(4):514–22. doi: 10.1093/heapol/czac005 (PMC9128743; doi:10.1093/heapol/czac005)
Supplement: czac005_Supp [file czac005_supp.zip › Supplimentary file 161221.docx]

**Supplement File: A systematic review of scope and quality of health economic evaluations conducted in Ethiopia**

**Appendix 1: PRISMA checklist**

| **Section/topic** | **#** | **Checklist item** | **Reported on page #** |
| --- | --- | --- | --- |
| **TITLE** | | |  |
| Title | 1 | Identify the report as a systematic review, meta-analysis, or both. | 1 |
| **ABSTRACT** | | |  |
| Structured summary | 2 | Provide a structured summary including, as applicable: background; objectives; data sources; study eligibility criteria, participants, and interventions; study appraisal and synthesis methods; results; limitations; conclusions and implications of key findings; systematic review registration number. | 1 |
| **INTRODUCTION** | | |  |
| Rationale | 3 | Describe the rationale for the review in the context of what is already known. | 2 |
| Objectives | 4 | Provide an explicit statement of questions being addressed with reference to participants, interventions, comparisons, outcomes, and study design (PICOS). | 3 |
| **METHODS** | | |  |
| Protocol and registration | 5 | Indicate if a review protocol exists, if and where it can be accessed (e.g., Web address), and, if available, provide registration information including registration number. | 3 |
| Eligibility criteria | 6 | Specify study characteristics (e.g., PICOS, length of follow-up) and report characteristics (e.g., years considered, language, publication status) used as criteria for eligibility, giving rationale. | 3, 4 |
| Information sources | 7 | Describe all information sources (e.g., databases with dates of coverage, contact with study authors to identify additional studies) in the search and date last searched. | 3-4 |
| Search | 8 | Present full electronic search strategy for at least one database, including any limits used, such that it could be repeated. | Supplimentary file |
| Study selection | 9 | State the process for selecting studies (i.e., screening, eligibility, included in systematic review, and, if applicable, included in the meta-analysis). | 3-4 |
| Data collection process | 10 | Describe method of data extraction from reports (e.g., piloted forms, independently, in duplicate) and any processes for obtaining and confirming data from investigators. | 4-6 |
| Data items | 11 | List and define all variables for which data were sought (e.g., PICOS, funding sources) and any assumptions and simplifications made. | 5, 6 |
| Risk of bias in individual studies | 12 | Describe methods used for assessing risk of bias of individual studies (including specification of whether this was done at the study or outcome level), and how this information is to be used in any data synthesis. | 5, 6 |
| Summary measures | 13 | State the principal summary measures (e.g., risk ratio, difference in means). | NA |
| Synthesis of results | 14 | Describe the methods of handling data and combining results of studies, if done, including measures of consistency (e.g., I^2^) for each meta-analysis. | NA |
| Risk of bias across studies | 15 | Specify any assessment of risk of bias that may affect the cumulative evidence (e.g., publication bias, selective reporting within studies). | NA |
| Additional analyses | 16 | Describe methods of additional analyses (e.g., sensitivity or subgroup analyses, meta-regression), if done, indicating which were pre-specified. | NA |
| **RESULTS** | | |  |
| Study selection | 17 | Give numbers of studies screened, assessed for eligibility, and included in the review, with reasons for exclusions at each stage, ideally with a flow diagram. | 6, 7 |
| Study characteristics | 18 | For each study, present characteristics for which data were extracted (e.g., study size, PICOS, follow-up period) and provide the citations. | 6 |
| Risk of bias within studies | 19 | Present data on risk of bias of each study and, if available, any outcome level assessment (see item 12). | 6, 7 |
| Results of individual studies | 20 | For all outcomes considered (benefits or harms), present, for each study: (a) simple summary data for each intervention group (b) effect estimates and confidence intervals, ideally with a forest plot. | Supplementary file |
| Synthesis of results | 21 | Present results of each meta-analysis done, including confidence intervals and measures of consistency. | 6-8 |
| Risk of bias across studies | 22 | Present results of any assessment of risk of bias across studies (see Item 15). | 8 |
| Additional analysis | 23 | Give results of additional analyses, if done (e.g., sensitivity or subgroup analyses, meta-regression [see Item 16]). | NA |
| **DISCUSSION** | | |  |
| Summary of evidence | 24 | Summarize the main findings including the strength of evidence for each main outcome; consider their relevance to key groups (e.g., healthcare providers, users, and policy makers). | 9-10 |
| Limitations | 25 | Discuss limitations at study and outcome level (e.g., risk of bias), and at review-level (e.g., incomplete retrieval of identified research, reporting bias). | 11 |
| Conclusions | 26 | Provide a general interpretation of the results in the context of other evidence, and implications for future research. | 12 |
| **FUNDING** | | |  |
| Funding | 27 | Describe sources of funding for the systematic review and other support (e.g., supply of data); role of funders for the systematic review. | Blinded title page |

**Appendix 2:** **ISPOR CiCERO Checklist:**

| **Evaluation approach:** | |
| --- | --- |
| Y = “Yes” or “Probably Yes”  N = “No”, “Probably No”, or “No Information”, unless the question specifies otherwise  NA = “Not Applicable” | |
| **General instructions:** |  |
| - Answer each question ONLY after providing answers to ALL the relevant sub-questions. - If **at least one** of the sub-questions is “No”, then answer “No”. - The questions answered as “NA” should be **excluded** from the grading. | |

| **Stage 1. Planning and development** | **Possible answers** |
| --- | --- |
|  | |
| **Question 1. Is the review conducted according to the predefined protocol?** | Y |
|  | |
| - 1. **Was evidence provided to document that the review methods were established *prior* to the conduct of the review?** | Y |
| ***Comment****:*   - *Answer “Yes” if the full-text protocol is accessible. (The review provides a link or a reference to the protocol.)* - *Answer “No” in all other cases.* | |
| - 1. **Did the review report whether there were any deviations from the protocol?** | Y |
| ***Comment:***   - *Answer “Yes” if the review had deviations from the protocol and reported them or the review reported that there were no deviations from the protocol* - *Answer “No” in the other cases* | |
| **Question 2. Does the review clearly report targeted population, outcomes, time horizon, study perspective, study design, and, when applicable, intervention(s) and comparator(s)?** | Y |
| ***Comment:***   - *Answer “Yes” for methodologic reviews, if at least the study design is reported.* - *Answer “No” in all other cases.* | |

| **Stage 2. Search for evidence** | **Possible answers** |
| --- | --- |
|  | |
| **Question 3. Did the review authors provide a detailed search strategy(-ies) for at least one database that includes the search month and year?** | Y |
| ***Comment***:   - *Answer “Yes” if the review authors provide the search strategy in either the main manuscript or an appendix AND report the search month and year.* - *Answer “No” in all other cases.* | |
| **Question 4. Is the search comprehensive and adequate?** | Y |
|  | |
| **4.1. Did the search include an argued range of databases / electronic sources for published literature relevant to the aim of the review?** | Y |
| ***Comment****:*   - *Answer “Yes” if a review has a global focus and includes more than two databases.* - *Answer “Yes” if a review has a regional/local focus, AND it includes both global and region-specific sources.* - *Answer “No” in all other cases.* | |
| **4.2. Was supplemental searching conducted to identify relevant reports for cost - or cost-effectiveness outcomes that were not identified in the database search(es)?** | Y |
| ***Comment****:*   - *Answer “Yes” if at least one additional method was used (eg tracking citations,* *consulting experts or searching relevant websites or references.)). See recommendations on supplementary literature searching.* - *Answer “NA” if review authors justify why supplementary search was not conducted.* - *Answer “No” in all other cases.* | |
| **4.3. Was a search for the relevant grey literature performed?** | Y |
| ***Comment****:*   - *Answer “Yes” if the review authors searched for grey literature relevant to the objective, (For example, did they search for HTA reports and/or scientific dissertations? See recommendations in subsection on grey literature search.)* - *Answer “NA” if the review makes a strong argument on why grey literature was not searched.* - *Answer “No” if the reviews did not search for the relevant grey literature or did not justify this decision.* - *Answer “No” in all other cases.* | |
| **4.4. Were the terms and structure of the search strategy sufficient to retrieve as many eligible studies as possible?** | Y |
| ***Comment:***   - *Answer “Yes” if the search terms were relevant to identify costs or cost-effectiveness studies. (See the recommendations in Stage 2.)* | |
| **Question 5. Were the search dates for the review provided? If “Yes”, was any justification for the search date provided?** | Y |
|  | |
| ***Comment:***   - *Answer “Yes” if the review reports the date range, the search dates and the reasons for dates ranges searched.* - *Answer “Yes” if the review provides the search dates while searching the evidence from commencement.* - *Answer “No” in all other cases.* |  |

| **Stage 3. Study selection and eligibility** | **Possible answers** |
| --- | --- |
|  | |
| **Question 6. Are the inclusion criteria relevant?** | Y |
|  | |
| **6.1. Did the review authors clearly report their inclusion criteria?** | Y |
|  | |
| **6.2. Are the inclusion criteria appropriate to answer the research question?** | Y |
|  | |
| **Question 7. Is the study selection process appropriate?** | Y |
|  | |
| **7.1. Did the review authors perform each step of the study selection independently in duplicate?** | Y |
| ***Comment:***   - *If not all of the steps of the selection process were performed in duplicate, say “Yes”* - *If review authors use the liberal accelerated approach in abstract screening and double reviewing in full-text screening, say “Yes”* - *If artificial intelligence is applied in the article search or screening, say “Yes” if the process was duplicated, and the review authors assess the possible biases by using this approach.* - *Answer “No” in all other cases.*   *See the recommendations on the screening approaches.* | |
| **7.2. If any restrictions to evidence inclusion were applied (ex. date, publication format or language), were they justified by the objectives of the review?** | NA |
| ***Comment:***   - *Answer “NA” if there were no restrictions mentioned.* - *Answer “Yes if a justification for restrictions was provided (eg, new technology, targeting the specific country or the region), or* - *Answer “Yes” if broad timeline restrictions are applied (>10 years).* - *Answer “No” in all other cases.* | |

| **Stage 4. Critical appraisal of included studies** | **Possible answers** |
| --- | --- |
|  | |
| **Question 8. Was an assessment of the methodological quality of included studies performed?** | Y |
| **Comment:**   - *Answer “Yes” if any peer-reviewed checklist (relevant to health economic studies) was used and reported to assess methodological quality in the original evidence. (See recommendations for the list of suggested instruments to use).* - *Answer “Yes” if no checklist was used, but the reviewers considered all important criteria (See Drummond and Jefferson (1996)^[[1]](#footnote-1)^ for the minimum necessary criteria).* - *Answer “Yes” if no studies were identified, but the methods section describes the methodological quality assessment approach in the manuscript or the protocol.*   *Answer “No” in all other cases (including when review authors state that they used the checklist, but don’t report the outcomes)* | |

| **Stage 5. Data extraction and synthesis** | **Possible answers** | |
| --- | --- | --- |
|  | | |
| **9. Was the data synthesized in a comprehensive, structured narrative way?** | | Y |
|  | | |

| Stage 6. Presentation and reporting | Possible answers | |
| --- | --- | --- |
|  | | |
| **Question 10. Were the original studies included in the review described in adequate detail?** | | Y |
| ***Comment:***  *Answer “NA” for each sub-question of question 10 if no studies were identified.* | | |
| **The reviews should report the following points for each of the included studies:** | |  |
| **10.1. Country of studied population** | | Y |
| **10.2. Description of the population of analysis** | | Y |
| **10.3. Time horizon, study perspective** | | Y |
| **10.4. Interventions compared** | | Y |
| ***Comment:*** *Answer “NA” if comparing interventions* *was not an objective of the review (eg, cost-of-illness/burden of disease)* | | |
| **10.5. Method(s) for valuation of economic outcomes** | | Y |
| **(a) Cost(s) in the health care sector according to the horizon of interest (direct costs, capital costs)** | | Y |
| **(b) Indirect medical costs** | | Y |
| **(c) Costs outside the healthcare sector such as productivity loss (indirect costs)** | | NA |
| **10.6. Method(s) for valuation of effectiveness outcomes, including source, type of source, estimates, duration (when relevant)** | | Y |
| ***Comment:*** *Answer “NA” if assessing cost-effectiveness was not an objective of the review (eg cost-minimization, cost-of-illness/burden of disease or other costs analysis).* | | |
| **10.7. Decision analytic modelling or approach to calculation of economic outcomes** | | Y |
| ***Comment:***  *Answer “NA” if the review includes only within-trial cost or cost-effectiveness studies.* | | |
| **10.8. Conflicts of interest and sources of funding** | | Y |
| **10.9. Software used (R, STATA, SAS, Excel, SPSS etc)** | | Y |

| **Question 11. Were the biases related to findings of the conducted review, including the conflicts of interest and funding of the reviewers, discussed?** | Y |
| --- | --- |

**Appendix 3a. Findings from cost-effectiveness analysis studies, N=26**

| Author, year | Perspective | Medical condition (type of intervention) | WTP used | Analysis/main findings | | Author’s conclusion |
| --- | --- | --- | --- | --- | --- | --- |
|  |  |  |  | **Base case analysis** | **Sensitivity analysis** |  |
| Accorsi et al, 2017 ^1^ | Healthcare provider | An ambulance-based referral system for emergency obstetrics and neonatal care | “acceptable” (<550), “attractive” (<150), and “very attractive” (<30) | US $24.7 per year life saved | The intervention remained “attractive” after a series of sensitivity analyses (e.g. costs of the ambulance referral system, the rate of effective referral). | An ambulance-based referral system for emergency obstetrics and neonatal care appears highly cost-effective. |
| Adelman et al 2017 ^2^ | Not mentioned | Xpert diagnostic algorithm plus WHO recommended TB symptom screen for TB case finding among PLHIV | US$ per DALY averted  WHO-CHOICE framework | ICER: US $5 per DALY averted | With Xpert sensitivity of 66%, SSX was both costlier and less effective than current recommended practice algorithm.  SSX was less cost-effective than base case with a high TB prevalence of 17% (ICER = $236 per DALY averted) | Combining a WHO recommended symptom screen with Xpert for TB diagnosis among PLHIV was highly cost-effective in a high-burden, resource-limited setting. |
| Alemayehu et al 2019 ^3^ | Healthcare provider | Managing MDR-TB at treatment initiative centre (TIC) vs treatment follow-up centre (TFC). | US$ per DALY averted  WHO-CHOICE framework | ICER of for TIC: US $1,641 per DALYs averted. | One way and Probabilistic sensitivity analysis were conducted.  The base case ICER finding was robust with a change in most of the parameters | Treatment of MDR-TB at both TIC and TFC are cost-effective interventions |
| Asebe et al 2021 ^4^ | Healthcare provider | Health extension program, multiple | US$ per LYG  WHO-CHOICE framework | ICER of US$21.60 to US$67.20 per LYG. | Discounting the LYG had the strong effect impact on the base case ICER | Health extension programs is very cost-effective in delivering community health services. |
| Belay et al 2021 | health care payer | HIV/AIDS  Dolutegravir‑ Versus Efavirenz‑Based Regimens as a First‑Line Treatment | US$ per QALY  WHO-CHOICE framework | Compared with the EFV-based regimen, DTG-based regimen was associated with an expected lifetime cost of $12,709 (vs. $12,701) and expected QALYs of 15.3 (vs. 14.7 QALYs) per patient  ICER: US$13.33 per QALY | DTG-based ART was dominant when 5-year time horizon was used.  All ICER values were below the estimated threshold value | DTG-based frst-line regimen appears to be cost efective compared with the EFV-based regimen for the treatment of HIV/AIDS patients in an Ethiopian setting |
| Bikilla et al 2009 ^5^ | Healthcare provider | ART for routine clinical practice in a district hospital setting in Ethiopia | WHO-CHOICE framework  GDP used: US $507 | ICER: US $314 per LYG | One way and probabilistic sensitivity analysis conducted.  Though the incremental cost per LYG under the assumption of reducing treatment effect of ART was considerably higher than that of the base case, all findings are less than three times the per capita GDP at the base year | ART could be regarded as cost-effective in a district hospital setting in Ethiopia |
| Carvalho et al 2020 ^6^ | Societal | Inhaled oxytocin product (IHO) for the prevention of Postpartum haemorrhage (PPH) compared to the standard of care in Bangladesh and Ethiopia | GDP used: US $768 (2017)  WHO-CHOICE framework | ICER: $1880 USD per maternal life year saved, | One way and probabilistic sensitivity analysis conducted.  The ICER of routine IHO administration considering recurring cost alone falls under 25% of per-capita GDP ($175 USD per maternal life-year saved) | As the ICER is 3 times the GDP, it is not cost-effective in Ethiopia |
| Cha et al 2020 ^7^ | Societal | Community-led total sanitation program | Not specified | Benefit–cost ratio was 3.7 and the net present value was international $1,193,786 | One-way and probabilistic sensitivity analyses were conducted.  The effect of the CLTS intervention and the assumed lifespan of an improved latrine were the main sources of sensitivity. | CLTS interventions can yield favourable economic returns, particularly if follow-up after the triggering is implemented intensively and uptake of improved latrines is achieved. |
| Crocker et al 2021 ^8^ | Societal | Community-led total sanitation program | Not specified | Cost effectiveness ranged from $34–$1897 per household ($5.85–$563 per person). | Cost effectiveness ration was sensitive to uncertainty about the costs and outcomes of the respective interventions. | For three out of four interventions, CLTS appeared more cost effective at reducing open defecation than at increasing latrine ownership, although sensitivity analysis revealed considerable variation. |
| Curry et al 2013 ^9^ | Not mentioned | The impact of the Ethiopian Millennium Rural Initiative (EMRI), 18-month systems-based intervention to improve the performance of 30 primary health care units in rural areas of Ethiopia. | GDP used: US $357  WHO-CHOICE framework | US $37,313 per life saved | The cost effectiveness of EMRI improves substantially if the performance achieved during the 18 months of the EMRI intervention is sustained for 5 years | The EMRI intervention, assuming only 18 months of improved health centre performance, is deemed to be not cost effective |
| Datik et al 2010 ^10^ | Societal | Involving health extension workers in TB treatment under a community-based initiative in Ethiopia (Health facility vs community based directly observed therapy; HFDOT vs CDOT) | Not mentioned | ICER of HFDOT to CDOT was -16.3. | One-way sensitivity  CDOT to be a more effective and less costly approach compared to HFDOT on varying estimates of the main cost item | Involving HEWs in TB treatment is a cost-effective treatment alternative to the health service and to the patients and their caregivers |
| Devine et al 2020 ^11^ | Healthcare provider | Sex-stratified plasmodium vivax treatment strategies using available G6PD diagnostics to accelerate access to radical cure | WHO-CHOICE framework | ICER for a sex-based treatment strategy with -day primaquine: $466 per DALY averted in Ethiopia | A one-way sensitivity analysis was conducted. A scenario analysis looked at the societal costs and varied efficacy estimates.  The different scenarios demonstrate roughly similar reductions in DALYs and similar costs associated with all three strategies | The treatment of G6PD normal females with high-dose 7-day primaquine regimen had the greatest impact on DALYs and was the only cost-effective option. |
| Eregata et al 2021 ^12^ | Health system | 159 health interventions used in the revision of Ethiopia’s essential health service package | GDP used: US $953 (2019 rate)  CHOICE framework | 95% of maternal health and infectious disease interventions had an ACER of less than US $1000 per HLY while 44% of non-communicable disease interventions had an ACER greater than US $1000 per HLY | No sensitivity analysis due to vast number of interventions | About 75% of the interventions evaluated had ACERs of less than US $1000 per HLY gained |
| Hailu et al 2018 ^13^ | Health provider | Combining long lasting insecticidal nets and indoor residual spraying compared for malaria prevention in Ethiopia | GDP used: US $628 (2014 rate)  CHOICE framework | ICER in the trial-based analysis: US $ −13,546 per DALY averted  ICER in Literature-based analysis: US $ 1403 per DALY averted | The annual malaria probability and protective effectiveness of combined intervention are key determinants of the cost-effectiveness of the interventions | Based on the current trial-based analysis, LLINs and IRS are not cost-effective compared to routine practice.  Based on the literature-based analysis, LLIN alone is likely to be cost-effective. |
| Hounsome et al 2019 ^14^ | Mixed (patient and programme implementation) | Hygiene and foot-care intervention for people with podoconiosis in the East Gojjam zone of northern Ethiopia | Not clear | Outcome measured by Dermatology Life Quality Index, Disability Assessment Schedule, and number of acute dermatolymphangioadenitis episode.  Intervention is cost saving compared to usual care. | Scenario analyses were conducted to account for training community podoconiosis assistants as a part of the intervention cost.  No pronounced effect on the results of the cost-effectiveness analysis. | Intervention is cost effective, more so for the poorest. |
| Jhons et al 2013 ^15^ | Not mentioned | Testing the impact of different degrees of antiretroviral therapy task shifting from physician to other health professionals in Ethiopia. | Not clear | For facilities with maximal task shifting, adjusted cost and proportion still active after 2 years is US $404 and 0.926 respectively. | Scenario analysis conducted.  Do significant difference in costs and outcome estimates | Maximal task shifting is more costly, less effective compared to minimal or moderate task shifting. |
| Kebede et al 2019 ^16^ | Societal | Introducing pneumococcal conjugate vaccine (PCV) 10 vaccination in the Ethiopian setting. | GDP used: US $505 (2013 rate)  WHO – CHOICE framework | ICER per averted DALY: US $ 413.8. | One-way sensitivity analysis was conducted.    ICER remained within 10% of the base case after ±20% changes in most parameters for the treatment and control groups. | General childhood PCV 10 vaccination was a cost-effective intervention |
| Kolesar et al 2017 ^17^ | Donor | Adding urine pregnancy test kits to the maternal and reproductive services package offered at the community level | WHO – CHOICE framework | ICER for the cost per life  US $2969.  Average cost per DALY averted (Ethiopia): US $47.95 | Monte Carlo simulations were conducted.  Higher mortality rates are associated with lower cost per death averted.  Increased uptake in short-acting hormonal family planning methods would reduce the cost per death averted. | The addition of urine pregnancy tests to an existing community health worker maternal and reproductive services package is highly cost-effective |
| Lemma et al 2011 ^18^ | Health provider | Comparing three different diagnosis and treatment strategies* | Not clear | ACER per correctly treated cases of parascreen pan/pf-based strategy was more cost-effective (US $1.69/CTC) than both the paracheck pf (US $4.66/CTC) and the presumptive (US $11.08/CTC) based strategies | One-way and two-way sensitivity analyses were conducted. And confirmed that parascreen pan/pf-based strategy as the most cost-effective in all scenarios. | The parascreen pan/pf-based strategy is cost-effective |
| Madan et al 2020 ^19^ | Health system | Switching to short treatment regimens for multidrug-resistant (MDR) tuberculosis | WTP thresholds: US $10,000 to US$ 100 000 | Health-care costs per participant per unfavourable outcome avoided in Ethiopia were US$ 6096.6 with the long and US$ 4552.3 for short regimen, respectively | Probabilistic sensitivity analysis revealed that the probability of short regimen being cost–effective declined as the value decision-makers placed on avoiding an unfavourable outcome increased. | The short MDR tuberculosis treatment regimen was associated with a substantial reduction in health-system costs and a lower financial burden for participants. |
| Mathewos et al 2017 ^20^ | Healthcare provider | Using Health Extension Workers (HEWs) for management of possible serious bacterial infection (PSBI) | GDP used: US $470 (2012 rate)  WHO – CHOICE framework | Cost per DALY averted: $223 | The high cost-effectiveness is dependent on PSBI management being added to an existing package of community-based maternal-newborn care | Adding PSBI management to a community-based maternal and newborn programme can be cost-effective |
| McPake et al 2015 ^21^ | Healthcare (government) provider | Implementing community-based practitioner programmes | WHO – CHOICE framework | ICER per life year gained was (in international dollars): $999 in Ethiopia | One-way and probabilistic sensitivity analysis were carried out.  The programmes are likely to be cost-effective (> 80% probability). | Community-based approaches are likely to be cost-effective for delivery of some essential health interventions where community-based practitioners operate within an integrated team supported by the health system |
| Memirie et al 2019 ^22^ | Healthcare provider | Selected Maternal and neonatal health (MNH) interventions | GDP used: US $707 (2016 rate)  WHO-CHOICE framework | Many of the MNH interventions analysed were highly cost-effective. | One-way and probabilistic sensitivity analysis were carried out, shich shows the substantial uncertainty that resides within the ICER estimates.  Despite variations, all the interventions except calcium supplementation. | 12 out of the 13 interventions included in our analysis were highly cost-effective.  Calcium supplementation does not appear to be cost-effective in our setting |
| Olsen et al 2021 ^23^ | Healthcare provider | Community-based treatment of childhood pneumonia | WHO-CHOICE framework | UD $ 15,000 per life saved and an increase in life expectancy at birth of 1.6 years across Ethiopia | Prioritizing regions with high under-five mortality rate are effective in reducing geographical inequalities, although at the cost of fewer lives saved as compared to the health maximizing strategy. | Making targeted efforts to scale-up the coverage in underprivileged regions first would prevent groups of people disadvantaged by residence from being left behind. |
| Strand et al 2016 ^24^ | Healthcare provider | Multiple neuropsychiatric interventions | WHO-CHOICE framework | Epilepsy with a first-generation antiepileptic drug: US$ 321 per DALY adverted).  Schizophrenia and bipolar disorders: US$ 1168–3739 per DALY adverted. | The size of the health budget has substantial impact on the certainty of the results | Epilepsy treatment is the most cost-effective whereas Schizophrenia and bipolar disorders are least cost-effective. |
| Tolla et al 2016 ^25^ | Healthcare provider | Prevention and treatment of ischemic heart disease (IHD) and stroke (15 single interventions and 16 intervention packages). | WHO-CHOICE framework | Combination drug treatment to individuals having an absolute risk >35 % yields the most value for money with an ICER of US $67 per DALY averted. | Cost-effectiveness ratios were relatively more sensitive to halving the effectiveness estimates as compared with doubling the price of drugs and laboratory tests | Primary prevention of IHD and stroke is a more efficient strategy for maximizing population-level health benefits compared with acute treatment and secondary prevention. |
| Yigezu et al 2020 ^26^ | Healthcare provider | Facility-based, stand-alone and mobile-based HIV voluntary counselling (VCT) and testing methods | WTP: US $220 to US $ 260 | Stand-alone-based VCT was extendedly dominated.  ICER for mobile-based VCT compared with facility-based VCT was USD 239 per HIV positive case. | One-way and probabilistic sensitivity analyses were conducted.  The cost of a positive test at mobile-based VCT had the highest impact on the ICER estimate | Using a mobile-based VCT approach costs less than both the facility-based and stand-alone approaches in terms of unit cost per tested individual and unit cost per HIV seropositive cases. Stand-alone-based VCT was not cost-effective |

**Appendix 3.b. Findings from extended cost-effectiveness analysis studies, N = 7**

| Author, year | Perspective | Medical condition (type of intervention) | Analysis/main findings (base case + sensitivity analysis) | Author’s conclusion |
| --- | --- | --- | --- | --- |
| Assebe et al 2020 ^27^ | Health system | Malaria  Publicly financing a 10% coverage increase in artemisinin-based combination therapy (ACT), long-lasting insecticide-treated bed nets (LLIN), indoor residual spraying (IRS), and malaria vaccine (hypothetical). | ACT, LLIN, IRS, and vaccine would avert 358, 188, 107 and 38 deaths, respectively, each year at a net government cost of $5.7, 16.5, 32.6, and 5.1 million, respectively.  The four interventions would eliminate about $4,627,800 of private health expenditures, | ACT, LLIN, IRS, and vaccine interventions would bring large health and financial benefits to the poorest households in Ethiopia. |
| Driessen et al 2015 ^28^ | Societal | Measles vaccination strategies  i) routine immunization,  ii) routine immunization with financial incentives, and  iii) mass campaigns – supplemental immunization activities (SIAs) | Deaths averted: 39,700 in SIAs), 10,300 in routine immunization with financial incentives and 4900 in the routine immunization without financial incentives.  The incentive option ($22,590,000) was estimated to increase costs ten-fold over the standard routine immunization offering ($2,158,000).  The most expensive undertaking was the SIAs, at over $23 million. | No one strategy was superior in terms of both health and economic benefits.  SIAs and routine immunization with financial incentives required similar levels of investment, with SIAs delivering a more sizable reduction in measles-related deaths and financial incentives |
| Johansson et al 2015 ^29^ | Health system | Pneumococcal Vaccination and Pneumonia Treatment | Scaling-up pneumococcal vaccines at around 40% coverage would cost about $11.5 million and avert about 2090 child deaths annually.  A 10% increase of pneumonia treatment to all children under 5 years of age would cost about $13.9 million and avert 2610 deaths annually.  The two interventions would eliminate a total of $2.4 million of private household expenditures annually | Pneumococcal vaccine and treatment interventions for children can bring large health and financial benefits to households in Ethiopia, most particularly among the poorest socio-economic groups. |
| Johansson et al 2017^30^ | Health provider | Mental Health Strategy (free of charge services for i) Epilepsy (75% coverage, phenobarbital).  ii) Depression (30% coverage, fluoxetine, cognitive therapy and proactive case management),  iii) Bipolar affective disorder (50% coverage, valproate and psychosocial therapy) and iv) Schizophrenia (75% coverage, haloperidol plus psychosocial treatment). | The package is expected to cost US$177 million and gain 155,000 HALYs (epilepsy US$37m and 64,500 HALYs; depression US$65m and 61,300 HALYs; bipolar disorder US$44m and 20,300 HALYs; and schizophrenia US$31m and 8,900 HALYs) annually. | The expected productivity gain is substantially higher than the expected financial risk protection |
| Pecenka et al 2014^31^ | Not clear | Diarrhoea  Universal public finance (UPF) of diarrhoeal treatment alone, as opposed to diarrhoeal treatment along with rotavirus vaccination in Ethiopia | Per US$1 million invested, diarrhoeal treatment saves 44 lives and averts US$115 000 in private expenditures. For the same investment, diarrhoeal treatment and rotavirus vaccination save 61 lives and avert US$150 000 in private expenditures. | Diarrhoeal treatment paired with rotavirus vaccination is more cost effective than diarrhoeal treatment alone |
| Shrime et al 2015^32^ | Not clear | Surgical access in rural Ethiopia  (a) universal public financing (UPF), which makes surgery free at the point of care but does not pay for non-medical costs,  (b) task sharing (TS) of surgery to non-surgeon providers, and  (c) a combination of UPF and TS,  (d) UPF with the addition of vouchers (UPFþV),  (e) TS plus V and  (f) UPF plus TS plus V. | Per million people per year in rural Ethiopia, UPF averted 23 deaths, at a cost of $945 000 (2 averted deaths per $100 000 spent, or $50 000/death averted).  TS was predicted to avert 253 deaths per million per year in rural Ethiopia, at a cost of $401 000 (64 averted deaths per $100 000, or $1500/death averted).  UPF + TS was predicted to cost the system $2 354 000 per million people per year, and to avert 289 deaths, for a total of 12 deaths averted per $100 000 spent ($8300/death). | Health benefits from each of the examined policies accrued primarily to the poor.  Without travel vouchers, many policies also induced impoverishment in the poor while providing financial risk protection to the rich. |
| Verguet et al 2015^33^ | Not clear | Public financing of selected interventions (measles vaccination, rotavirus vaccination, pneumococcal conjugate vaccination, diarrhoea treatment, malaria treatment, pneumonia treatment, caesarean section surgery, hypertension treatment, and tuberculosis treatment.) | Per dollar spent by the Ethiopian Government, the interventions that avert the most deaths are measles vaccination (367 deaths averted per $100 000 spent), pneumococcal conjugate vaccination (170 deaths averted per $100 000 spent), and caesarean section surgery (141 deaths averted per $100 000 spent).  The interventions that avert the most cases of poverty are caesarean section surgery (98 cases averted per $100 000 spent), tuberculosis treatment (96 cases averted per $100 000 spent), and hypertension treatment (84 cases averted per $100 000 spent). |  |

**Appendix 4a.** Search strategy for PubMed search engine.

| ((((((((((((((((((((((((((((((((((((((((((((((((((Costs, Cost Analysis) OR Cost, Cost Analysis) OR ((Costs and Cost Analyses))) OR Cost Analysis) OR Cost Analyses) OR Analysis, Cost) OR Cost Comparison) OR Comparison, Cost) OR Comparisons, Cost) OR Cost Comparisons) OR Cost-Minimizaon Analysis) OR Analyses, Cost-Minimizaon) OR Analysis, Cost-Minimizaon) OR Cost Minimizaon Analysis) OR Cost-Minimizaon Analyses) OR Pricing) OR Cost) OR Costs) OR Illness Cost) OR Cost of Disease) OR Costs, Disease) OR Costs, Sickness) OR Economic Burden of Disease) OR Analyses, CostBenefit) OR Analysis, Cost-Benefit) OR Analyses, Cost Benefit) OR Analysis, Cost Benefit) OR Cost Effecveness) OR Effecveness, Cost) OR Cost-Ulity Analysis) OR Cost Benefit Data) OR Analyses, Cost-Ulity) OR Analysis, Cost-Ulity) OR Cost Ulity Analysis) OR Cost-Ulity Analyses) OR Economic Evaluaon) OR Economic Evaluaons) OR Evaluaon, Economic) OR Evaluaons, Economic) OR Marginal Analysis) OR Analyses, Margina) OR analysis,marginal) OR Analysis, Marginal) OR Marginal Analyses) OR Cost Benefit) OR ((Costs and Benefits))) OR ((Benefits and Costs))) AND "Ethiopia"[Mesh]))). |
| --- |

**Appendix 4b.** list of the websites searched for studies.

| **Name** | **Website** |
| --- | --- |
| Disease Control Priorities-Ethiopia | <http://dcp-3.org/country-work/ethiopia> |
| WHO-CHOICE (CHOosing Interventions that are Cost-Effective) program | <https://www.who.int/teams/health-systems-governance-and-financing/economic-analysis> |
| Ministry of Health Ethiopia | <https://www.moh.gov.et/> |
| CEA Tufts | <https://cevr.tuftsmedicalcenter.org/databases/cea-registry> |

**References**

1. Accorsi S, Somigliana E, Solomon H, et al. Cost-effectiveness of an ambulance-based referral system for emergency obstetrical and neonatal care in rural Ethiopia. *BMC pregnancy and childbirth.* 2017;17(1):1-7.

2. Adelman MW, McFarland DA, Tsegaye M, Aseffa A, Kempker RR, Blumberg HM. Cost-effectiveness of WHO-Recommended Algorithms for TB Case Finding at Ethiopian HIV Clinics. Paper presented at: Open forum infectious diseases2018.

3. Alemayehu S, Yigezu A, Hailemariam D, Hailu A. Cost-effectiveness of treating multidrug-resistant tuberculosis in treatment initiative centers and treatment follow-up centers in Ethiopia. *Plos one.* 2020;15(7):e0235820.

4. Assebe LF, Belete WN, Alemayehu S, et al. Economic evaluation of Health Extension Program packages in Ethiopia. *Plos one.* 2021;16(2):e0246207.

5. Bikilla AD, Jerene D, Robberstad B, Lindtjørn B. Cost-effectiveness of anti-retroviral therapy at a district hospital in southern Ethiopia. *Cost Effectiveness and Resource Allocation.* 2009;7(1):1-11.

6. Carvalho N, Hoque ME, Oliver VL, et al. Cost-effectiveness of inhaled oxytocin for prevention of postpartum haemorrhage: a modelling study applied to two high burden settings. *BMC medicine.* 2020;18(1):1-18.

7. Cha S, Jung S, Bizuneh DB, et al. Benefits and Costs of a Community-Led Total Sanitation Intervention in Rural Ethiopia—A Trial-Based Ex Post Economic Evaluation. *International journal of environmental research and public health.* 2020;17(14):5068.

8. Crocker J, Fuente D, Bartram J. Cost effectiveness of community led total sanitation in Ethiopia and Ghana. *International journal of hygiene and environmental health.* 2021;232:113682.

9. Curry LA, Byam P, Linnander E, et al. Evaluation of the Ethiopian Millennium Rural Initiative: impact on mortality and cost-effectiveness. *PLoS One.* 2013;8(11):e79847.

10. Datiko DG, Lindtjørn B. Cost and cost-effectiveness of treating smear-positive tuberculosis by health extension workers in Ethiopia: an ancillary cost-effectiveness analysis of community randomized trial. *PLoS One.* 2010;5(2):e9158.

11. Devine A, Howes RE, Price DJ, et al. Cost-Effectiveness Analysis of Sex-Stratified Plasmodium vivax Treatment Strategies Using Available G6PD Diagnostics to Accelerate Access to Radical Cure. *The American journal of tropical medicine and hygiene.* 2020;103(1):394-403.

12. Eregata GT, Hailu A, Stenberg K, Johansson KA, Norheim OF, Bertram MY. Generalised cost-effectiveness analysis of 159 health interventions for the Ethiopian essential health service package. *Cost Effectiveness and Resource Allocation.* 2021;19(1):1-13.

13. Hailu A, Lindtjørn B, Deressa W, Gari T, Loha E, Robberstad B. Cost-effectiveness of a combined intervention of long lasting insecticidal nets and indoor residual spraying compared with each intervention alone for malaria prevention in Ethiopia. *Cost Effectiveness and Resource Allocation.* 2018;16(1):1-17.

14. Hounsome N, Kassahun MM, Ngari M, et al. Cost-effectiveness and social outcomes of a community-based treatment for podoconiosis lymphoedema in the East Gojjam zone, Ethiopia. *PLoS neglected tropical diseases.* 2019;13(10):e0007780.

15. Johns B, Asfaw E, Wong W, et al. Assessing the costs and effects of antiretroviral therapy task shifting from physicians to other health professionals in ethiopia. *JAIDS Journal of Acquired Immune Deficiency Syndromes.* 2014;65(4):e140-e147.

16. Kebede TT, Svensson M, Addissie A, Trollfors B, Andersson R. Cost-effectiveness of childhood pneumococcal vaccination program in Ethiopia: results from a quasi-experimental evaluation. *BMC public health.* 2019;19(1):1-12.

17. Kolesar RJ, Audibert M, Comfort AB. Cost-effectiveness analysis and mortality impact estimation of scaling-up pregnancy test kits in Madagascar, Ethiopia and Malawi. *Health policy and planning.* 2017;32(6):869-881.

18. Lemma H, San Sebastian M, Löfgren C, Barnabas G. Cost-effectiveness of three malaria treatment strategies in rural Tigray, Ethiopia where both Plasmodium falciparum and Plasmodium vivax co-dominate. *Cost effectiveness and resource allocation.* 2011;9(1):1-9.

19. Madan JJ, Rosu L, Tefera MG, et al. Economic evaluation of short treatment for multidrug-resistant tuberculosis, Ethiopia and South Africa: the STREAM trial. *Bulletin of the World Health Organization.* 2020;98(5):306.

20. Mathewos B, Owen H, Sitrin D, et al. Community-Based interventions for newborns in Ethiopia (combine): cost-effectiveness analysis. *Health policy and planning.* 2017;32(suppl_1):i21-i32.

21. McPake B, Edoka I, Witter S, et al. Cost-effectiveness of community-based practitioner programmes in Ethiopia, Indonesia and Kenya. *Bulletin of the World Health Organization.* 2015;93:631-639.

22. Memirie ST, Tolla MT, Desalegn D, et al. A cost-effectiveness analysis of maternal and neonatal health interventions in Ethiopia. *Health policy and planning.* 2019;34(4):289-297.

23. Olsen M, Norheim OF, Memirie ST. Reducing regional health inequality: a sub-national distributional cost-effectiveness analysis of community-based treatment of childhood pneumonia in Ethiopia. *International Journal for Equity in Health.* 2021;20(1):1-10.

24. Strand KB, Chisholm D, Fekadu A, Johansson KA. Scaling-up essential neuropsychiatric services in Ethiopia: a cost-effectiveness analysis. *Health policy and planning.* 2016;31(4):504-513.

25. Tolla MT, Norheim OF, Memirie ST, et al. Prevention and treatment of cardiovascular disease in Ethiopia: a cost-effectiveness analysis. *Cost Effectiveness and Resource Allocation.* 2016;14(1):1-14.

26. Yigezu A, Alemayehu S, Hamusse SD, Ergeta GT, Hailemariam D, Hailu A. Cost-effectiveness of facility-based, stand-alone and mobile-based voluntary counseling and testing for HIV in Addis Ababa, Ethiopia. *Cost Effectiveness and Resource Allocation.* 2020;18(1):1-12.

27. Assebe LF, Kwete XJ, Wang D, et al. Health gains and financial risk protection afforded by public financing of selected malaria interventions in Ethiopia: an extended cost-effectiveness analysis. *Malaria journal.* 2020;19(1):41.

28. Driessen J, Olson ZD, Jamison DT, Verguet S. Comparing the health and social protection effects of measles vaccination strategies in Ethiopia: An extended cost-effectiveness analysis. *Social Science & Medicine.* 2015;139:115-122.

29. Johansson KA, Memirie ST, Pecenka C, Jamison DT, Verguet S. Health gains and financial protection from pneumococcal vaccination and pneumonia treatment in Ethiopia: results from an extended cost-effectiveness analysis. *PloS one.* 2015;10(12):e0142691.

30. Johansson KA, Strand KB, Fekadu A, Chisholm D. Health gains and financial protection provided by the Ethiopian mental health strategy: an extended cost-effectiveness analysis. *Health Policy and Planning.* 2017;32(3):376-383.

31. Pecenka CJ, Johansson KA, Memirie ST, Jamison DT, Verguet S. Health gains and financial risk protection: an extended cost-effectiveness analysis of treatment and prevention of diarrhoea in Ethiopia. *BMJ open.* 2015;5(4):e006402.

32. Shrime MG, Verguet S, Johansson KA, Desalegn D, Jamison DT, Kruk ME. Task-sharing or public finance for the expansion of surgical access in rural Ethiopia: an extended cost-effectiveness analysis. *Health policy and planning.* 2016;31(6):706-716.

33. Verguet S, Murphy S, Anderson B, Johansson KA, Glass R, Rheingans R. Public finance of rotavirus vaccination in India and Ethiopia: an extended cost-effectiveness analysis. *Vaccine.* 2013;31(42):4902-4910.

Supplementary figure 1. Percent of studies that fulfilled the item criteria, N=27


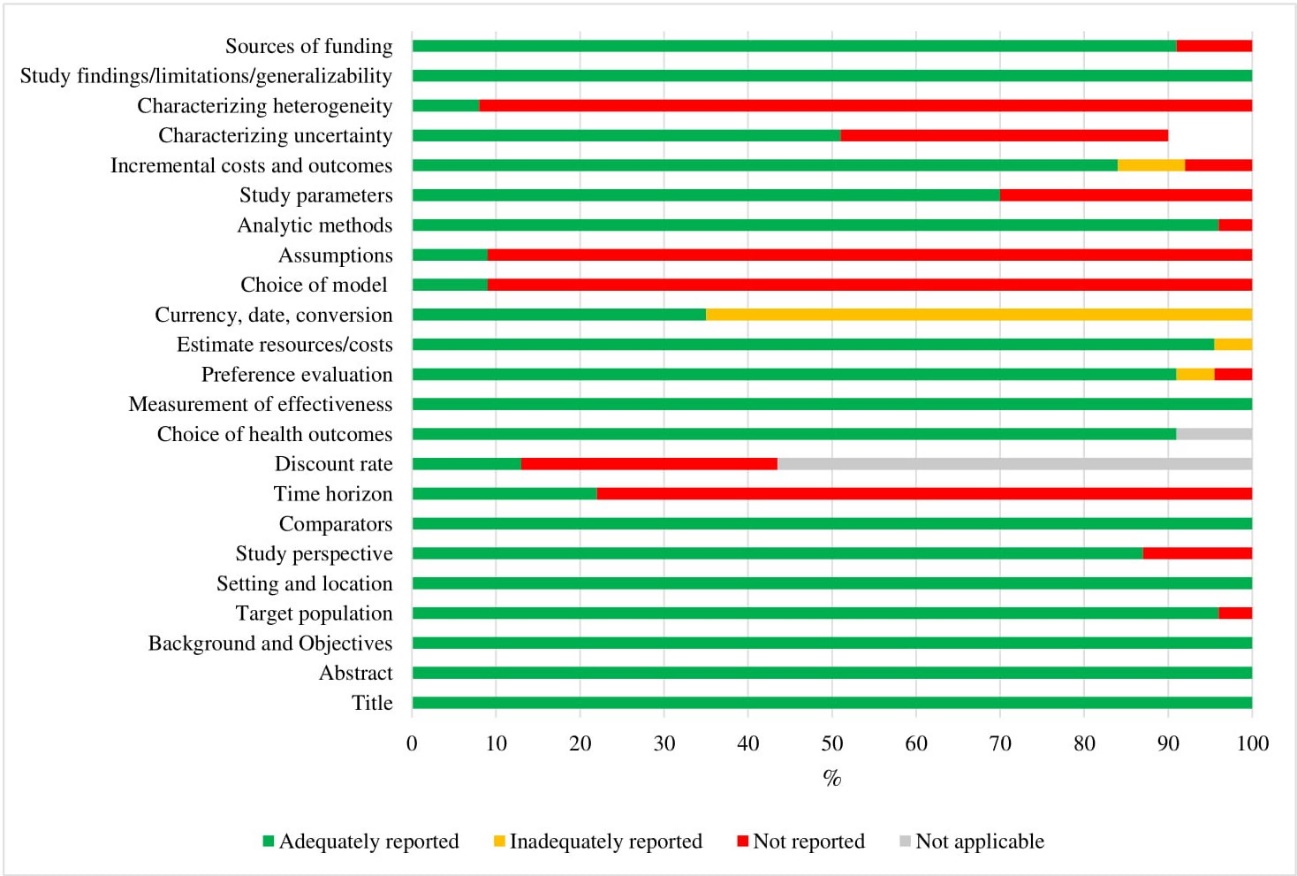


1. Drummond MF, Jefferson TO. Guidelines for authors and peer reviewers of economic submissions to the BMJ. The BMJ Economic Evaluation Working Party. *BMJ*. 1996;313:275-83. [↑](#footnote-ref-1)
